# Contralateral Neurovascular Coupling in Patients with Ischemic Stroke After Endovascular Thrombectomy

**DOI:** 10.1007/s12028-024-02178-w

**Published:** 2025-01-07

**Authors:** Zhe Zhang, Shafiul Hasan, Ofer Sadan, Eric S. Rosenthal, Yuehua Pu, Zhixuan Wen, Changgeng Fang, Xin Liu, Wanying Duan, Liping Liu, Ran Xiao, Xiao Hu

**Affiliations:** 1 Neurocritical Care Unit, Department of Neurology, Beijing Tiantan Hospital, Capital Medical University, 119 South 4th Ring Rd W, Beijing, 100070 China; 2 Center for Data Science, Nell Hodgson Woodruff School of Nursing, Emory University, Atlanta, GA USA; 3 Division of Neurocritical Care, Department of Neurology, Emory University School of Medicine, Atlanta, GA USA; 4 Neurosciences Intensive Care Unit, Massachusetts General Hospital, Boston, MA USA

**Keywords:** Neurovascular coupling, Ischemic stroke, Collateral circulation, Cerebrovascular circulation, Cerebral revascularization

## Abstract

**Background:**

Neurovascular coupling (NVC) refers to the process of aligning cerebral blood flow with neuronal metabolic demand. This study explores the potential of contralateral NVC—linking neural electrical activity on the stroke side with cerebral blood flow velocity (CBFV) on the contralesional side—as a marker of physiological function of the brain. Our aim was to examine the association between contralateral NVC and neurological outcomes in patients with ischemic stroke following endovascular thrombectomy.

**Methods:**

We concurrently recorded the CBFVs of the middle cerebral arteries and electroencephalographic (EEG) signals of patients after endovascular thrombectomy. We employed phase-amplitude cross-frequency coupling to quantify the contralateral coupling between EEG activity on the stroke side and CBFV on the contralesional side. Key neurological outcomes were measured, including changes in National Institute of Health Stroke Scale (NIHSS) scores, infarct volume progression over 7 days, and modified Rankin Scale scores at 90 days.

**Results:**

A total of 52 study participants were enrolled in our study (mean age 61.5 ± 10.4 years; 90.4% male; median preprocedural NIHSS score 14 [interquartile range 10–17]). We successfully computed contralateral NVC in 48 study participants. A significant association emerged between contralateral coupling and improvements in NIHSS scores over 7 days (theta band, *P* = 0.030) and in infarct volume progression (delta band, *P* = 0.001; theta band, *P* = 0.013). Stronger contralateral NVC in the delta and theta bands correlated with better outcomes at 90 days (adjusted odds ratio for delta 7.53 [95% confidence interval 1.13–50.30], *P* = 0.037; adjusted odds ratio for theta 6.36 [95% confidence interval 1.09–37.01], *P* = 0.039).

**Conclusions:**

A better contralateral coupling between stroke-side EEG and contralesional CBFV is associated with favorable neurological outcomes, suggesting that contralateral NVC analysis may aid in assessing brain function after recanalization. Replication with a deeper understanding of the mechanisms is needed before clinical translation.

**Supplementary Information:**

The online version contains supplementary material available at 10.1007/s12028-024-02178-w.

## Introduction

Several randomized controlled trials have provided robust evidence that endovascular thrombectomy (EVT) improves outcomes for patients with ischemic stroke due to large vessel occlusions [1–4]. Nevertheless, the prognosis of nearly half of patients after EVT remains unfavorable [1, 5]. Recent studies indicated that microcirculatory failure resulted in the “no-reflow” phenomenon, in which brain tissue remained ischemic despite macrovascular recanalization [6, 7]. It remains unclear whether the reperfused hemisphere still desires more blood, specifically supplementary blood flow from the contralesional side.

The brain’s high metabolic demands are challenged by limited energy storage capacity. Neurovascular coupling (NVC) is a mechanism that rapidly enables cerebral blood flow (CBF) to match the neuronal metabolic demand [8]. Impaired NVC has been found in various clinical conditions, including stroke, traumatic brain injury, and cognitive disorders [9]. Early after recanalization, assessing NVC could potentially aid in evaluating patient prognosis.

Traditional methods for assessing NVC, such as functional magnetic resonance imaging (MRI) and transcranial Doppler (TCD), measure increased CBF in response to task or stimuli [10]. However, these methods bypass the step to prove a stimulus induces a change in neuronal electrical activity. In other words, they are unable to link the electrical activities in specific cortical regions to the source of altered CBF. To address this limitation, a novel algorithm has been developed for calculating the coupling index between electroencephalographic (EEG) signals and CBF velocity (CBFV) at resting state [11, 12]. Using this algorithm, we cannot only explore the coupling between lesional-side EEG and ipsilateral CBF (ipsilateral NVC), but also quantify how the contralesional CBF responds to lesional-side EEG (contralateral NVC).

Our aim is to investigate whether the salvaged brain tissue still requires supplementary blood flow from the contralesional side in cases in which an occluded large vessel has achieved successful recanalization. Specifically, we seek to quantitatively assess the impact of contralateral NVC on the prognosis. Our hypothesis was that impaired contralateral NVC may be associated with poor outcomes after EVT. In this study, we calculated contralateral NVC metrics to test this hypothesis.

## Methods

### Study Design and Study Participants

This was a single-center, retrospective, observational study. The clinical and neuroimaging data were obtained from a prospectively collected cohort (Registry for Critical Care of Acute Stroke in China, registered at www.chictr.org.cn, identifier ChiCTR1900022154). Patients with large vessel occlusive (LVO) stroke, which included the middle cerebral artery (MCA), who had undergone EVT in Beijing Tiantan Hospital, aged 18–85 years, and premorbid modified Rankin Scale (mRS) 0–2, were eligible to enroll. We excluded study participants with infarcts within vertebrobasilar artery territory, those with bilateral hemisphere infarcts, and those with severe systemic medical comorbidities or terminal illness with an anticipated life expectancy of less than one year (including conditions such as congestive heart failure [New York Heart Association Class IV], hepatic failure, and malignant tumors). We also excluded those enrolled in other clinical trials, those with poor acoustic temporal windows, and patients who could not tolerate the EEG-TCD monitoring due to delirium, or those requiring sedation or anesthesia during data acquisition. Management decisions adhered to the latest relevant guidelines available at the time of patient enrollment [13, 14]. The Ethics Committee of Beijing Tiantan Hospital approved the study. Written informed consent was obtained from the legally authorized representatives for all individual participants included in the study.

### EEG-TCD Monitoring

**Fig. 1:**
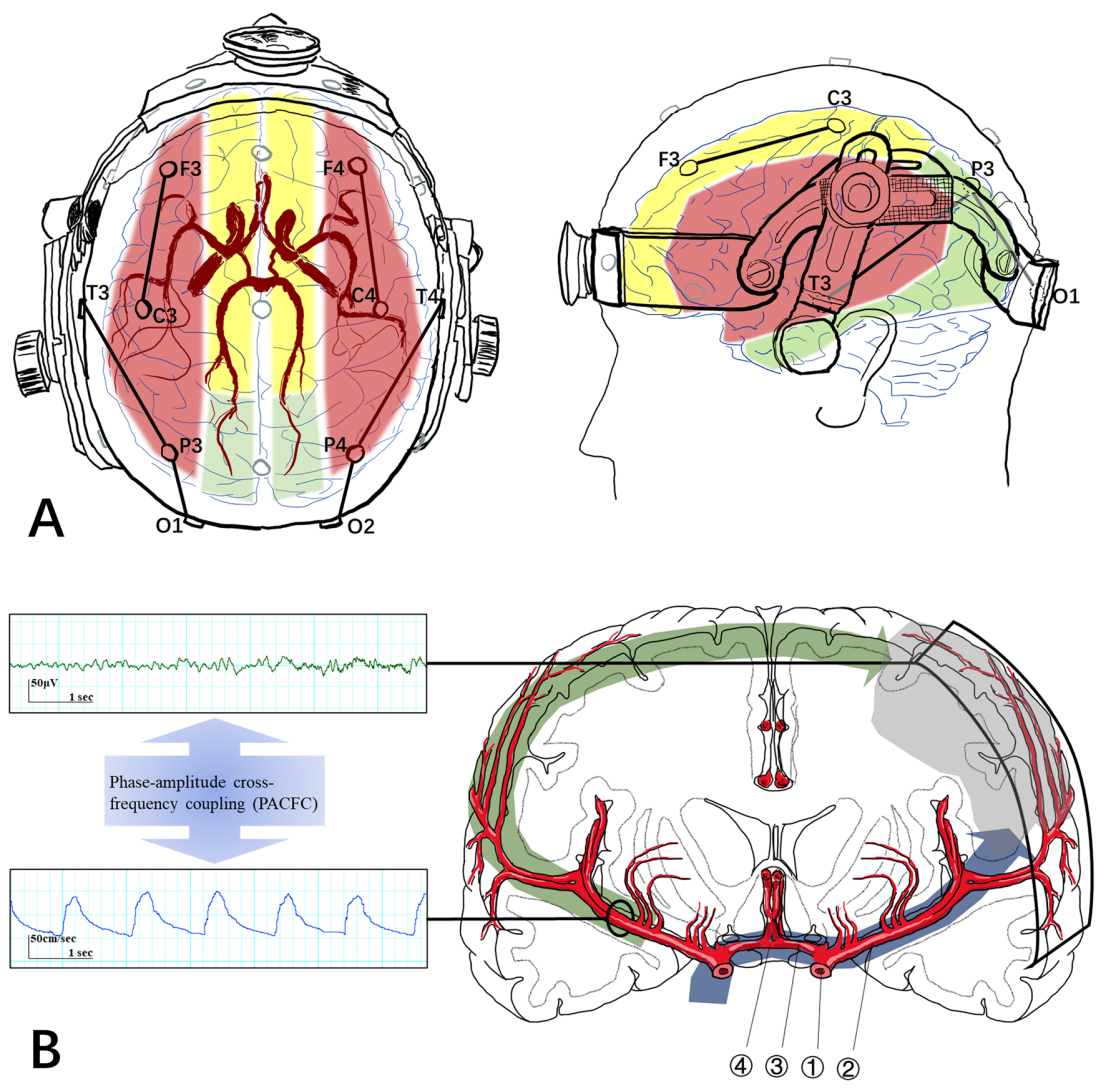
Data acquisition and contralateral neurovascular coupling. **a** We synchronously recorded the CBFV of MCA and the EEG signals from the brain region supplied by MCA. The TCD probes were placed at the temporal windows, with a measurement depth of 50–65 mm, reflecting the M1 segment of MCA. We selected three pairs of bipolar channels (F3-C3/F4-C4, T3-P3/T4-P4, and P3-O1/P4-O2) to cover the MCA territory (red area). The ACA and PCA territory were represented by yellow and green areas, respectively. **b** PACFC was used to bridge the EEG from the stroke-side hemisphere and CBFV of the contralesional MCA to calculate the contralateral NVC. Bilateral A1 segments of ACA (③) and ACoA (④) forms the aCoW, which serves as the primary collateral connecting bilateral ICA (①) and MCA (②). Of note, the aCoW is not always complete. As shown in the figure, cerebral blood flow signal was recorded in the M1 segment of MCA located at the distal to ICA. Therefore, the contralateral NVC in this study was unlikely to represent the primary collateral through the aCoW (blue pathway), but rather reflected collaterals via the leptomeningeal and other anastomoses (green pathway). ACA, anterior cerebral artery, ACoA, anterior communicating artery, aCOW, anterior Circle of Willis, CBFV, cerebral blood flow velocity, EEG, electroencephalography, ICA, internal carotid artery, MCA, middle cerebral artery, NVC, neurovascular coupling, PACFC, phase-amplitude cross-frequency coupling, PCA, posterior cerebral artery (Color figure online).

Patients were monitored synchronously with EEG (Nicolet, Natus Medical, USA), TCD (DWL, Compumedics DWL, Germany), and arterial blood pressure in the neurocritical care unit setting within 48 h after EVT. The patients lay quietly in bed, and no stimulation or task was applied during the monitoring. EEG electrodes were placed on the scalp according to the international 10–20 system. To represent the cerebral regions supplied by MCAs with fewer electrodes, three pairs of bipolar channels (F3-C3/F4-C4, T3-P3/T4-P4, and P3-O1/P4-O2) were selected for analyzing the EEG signal. Two 2.5-MHz TCD transducers, fitted on a headband, were placed over the temporal window. The depth of insonation was adjusted between 50 and 65 mm to measure the CBFV of bilateral MCA (Fig. 1a).

### EEG-TCD Data Processing and Calculation

Phase-amplitude cross-frequency coupling (PACFC) was used to assess the contralateral coupling between EEG amplitude from the stroke hemisphere and CBFV of the contralesional MCA (Fig. 1b). First, artifact-free data were manually selected, and CBFV was filtered (0.05–0.15 Hz) to reduce respiratory-related fluctuations. Using Hilbert transform, we extracted EEG amplitude and CBFV phase. The modulation index was calculated to quantify coupling, with high modulation index indicating strong neurovascular coupling. Surrogate data generated a null distribution, fitting a Gaussian to obtain the mean (*μ*) and standard deviation (*σ*). Normalized PACFC was computed for statistical analysis. Fifty 5-min data segments were analyzed per participant, considering EEG frequency bands (delta, theta, alpha, beta) and other metrics. The detailed process of calculation was presented in our previous work [11] and the supplementary materials.

To better understand the differences in PACFC values across frequency bands, and exclude possible confounding factors, we also calculated the metrics including ipsilateral PACFC values, alpha to delta power ratio (ADR), and mean velocity index (Mx) (a cerebral autoregulation parameter) on stroke and contralesional sides [15]. All the computations were performed using Matlab 2022b (MathWorks, Massachusetts, USA) and Python 3.12.

### Outcomes

The National Institute of Health Stroke Scale (NIHSS) score at admission to the hospital and at 7 days (or at discharge from the neurocritical care unit, if earlier than 7 days), along with mRS scores at 90 ± 7 days were collected by investigators independent of the EEG-TCD monitoring. We defined ΔNIHSS (ΔNIHSS = NIHSS scores at 7 days − NIHSS scores at admission) to represent early neurological function changes. mRS score 0–2 at 90 days was regarded as a favorable outcome.

All patients underwent computed tomography (CT) angiography and perfusion imaging, or MRI scanning, at baseline and 24 h after the EVT procedure. A plain CT was performed at 7 days and whenever necessary thereafter. The infarct size before the procedure and at 7 days were estimated by RAPID software (iSchemaView, California, USA) and manual measurement by independent neuroimaging raters, respectively. Infarct growth was defined as the difference between the infarct volume at 7 days and the baseline.

To investigate how the completeness of the anterior Circle of Willis (aCoW) affects the contralateral coupling, we further performed subgroup analysis by dichotomizing the study participants into complete and incomplete aCoW subgroups. The aCoW was incomplete if the anterior communicating artery or A1 segment(s) of anterior cerebral artery (ACA) was hypoplastic or absent in either hemisphere [16] as determined by digital subtraction angiography or CT angiography.

### Statistical Analysis

Continuous variables were summarized as means ± standard deviation for normally distributed data, and medians with interquartile ranges (IQRs) for nonnormally distributed data. Categorical variables were reported in numbers (%). We used *t*-test, *χ*^2^ test, Fisher’s exact test, and Wilcoxon rank-sum tests as appropriate for unadjusted comparisons. The Spearman correlation coefficient was applied to examine the correlation between the two groups. Odds ratios (ORs) and 95% confidence intervals (CIs) of dichotomous variables were estimated using a logistic regression model. We standardized the variables into normal distribution to avoid the unproportionally impact from variables with large magnitude on the regression model. In the multivariable regression, we accounted for known predictors of outcome after LVO stroke, including age, preprocedural NIHSS score, ASPECTS, 24-h stroke volume, and modified Thrombolysis in Cerebral Infarction (mTICI) score. Statistical analyses were performed using StataMP 17.0 (Stata Corporation, Texas, USA). *P* values < 0.05 were considered statistically significant.

## Results

**Fig. 2:**
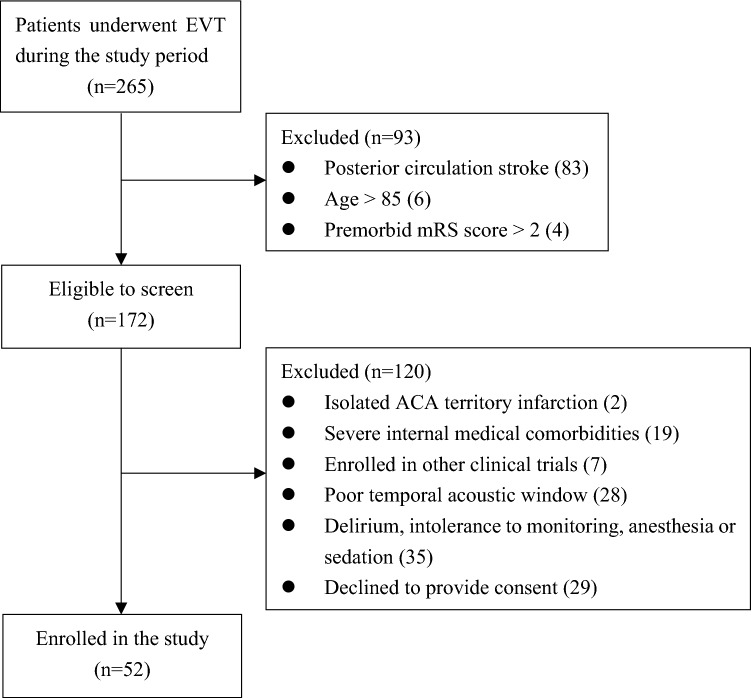
Flowchart of the study. ACA, anterior cerebral artery, EVT, endovascular thrombectomy, mRS, modified Rankin Scale

Between June 2017 and May 2019, a total of 52 patients with LVO stroke were enrolled in this research (Table 1, Fig. 2). A total of 90.4% (47/52) of patients achieved successful recanalization (mTICI score 2b–3). A total of 73.1% (38/52) of patients received oral and/or intravenous antihypertensive medications. None of the study participants were prescribed vasopressors. The EEG-TCD monitoring began at 30.0 ± 14.9 h after EVT, with 2.1 ± 0.8 h of recording. Four patients were excluded from the PACFC calculation because of poor signal quality. The ipsilateral coupling index on the stroke side showed median values (with IQRs) of delta 2.65 (2.17–3.31), theta 2.86 (2.31–3.35), alpha 2.77 (2.40–3.47), and beta 3.14 (2.45–3.96), whereas on the contralesional side, the values were 2.74 (2.42–3.06), 2.89 (2.25–3.45), 2.81 (2.36–3.66), and 2.91 (2.36–3.73), respectively. These differences across frequency bands between the stroke and contralesional sides were not statistically significant at 95% significance level. The medians (IQRs) of contralateral coupling index of delta, theta, alpha, and beta EEG frequency bands were 2.63 (2.34–3.25), 2.77 (2.46–3.43), 2.73 (2.44–3.40), and 2.96 (2.61–3.95), respectively. The contralateral coupling index across four frequency bands were not associated with age (*P* values for delta, theta, alpha, and beta bands were 0.459, 0.507, 0.462, and 0.587). No significant differences in contralateral coupling index across four frequency bands were found between patients with or without mechanical ventilation or antihypertensive treatment (all *P* > 0.05). ADR of the stroke-affected hemisphere was significantly lower than that of the contralesional side (1.13 [0.64–2.04] versus 2.10 [1.04–3.46], *P* = 0.01), whereas the Mx between the stroke and the contralesional sides were not statistically different (0.0443 [0.0171–0.0815] versus 0.036 [0.0144–0.0639], *P* = 0.333).

**Table 1:** Patient characteristics

Variables	mean ± SD, median (IQR), or n (%)
Age, yr, mean ± SD	61.5 ± 10.4
Male, n (%)	47 (90.4)
Clinical, laboratory, and imaging data at admission to the hospital	
Preprocedural NIHSS, median (IQR)	14 (10–17)
Admission SBP mm Hg, mean ± SD	151.5 ± 24.9
Admission DBP mm Hg, mean ± SD	86.3 ± 15.7
Admission glucose, mmol/L, mean ± SD	8.2 ± 2.8
Left-sided infarct, n (%)	31 (59.6)
ASPECTS score, median (IQR)	9 (8–9)
Preprocedural infarct size, mL, median (IQR)	8.5 (4.0–26.4)
Occlusion on angiography, n (%)	
ICA (C1–3)	22 (42.3)
ICA (C4–7)	9 (17.3)
MCA (M1–M2)	21 (40.4)
ASITN/SIR collateral grading scale score, n (%)	
0	1 (1.9)
1	32 (61.5)
2	16 (30.8)
3	3 (5.8)
4	0
Medical history, n (%)	
Hypertension	34 (65.4)
Coronary artery disease	11 (21.2)
Atrial fibrillation	15 (28.8)
Dislipidemia	17 (32.7)
Diabetes mellitus	21 (40.4)
Smoking	27 (51.9)
TIA or stroke	21 (40.4)
Stroke subtype, n (%)	
Large artery atherosclerotic	38 (73.1)
Cardioembolism	14 (26.9)
Recanalization treatment	
Intravenous tPA, n (%)	18 (34.6)
Time from symptom onset to reperfusion, min, median (IQR)	530 (420–700)
mTICI score, n (%)	
0	4 (7.7)
1	0 (0)
2a	1 (1.9)
2b	13 (25.0)
3	34 (65.4)
Treatment in NeuroICU	
Mechanical ventilation	12 (23.1)
Oral antihypertensive therapy	22 (42.3)
IV antihypertensive therapy	32 (61.5)
Hemorrhagic transformation, n (%)	
HI 1	3 (5.8)
HI 2	11 (21.2)
PH 1	1 (1.9)
PH 2	5 (9.6)
SICH	3 (5.8)
Infarct growth at 7 days, mL, median (IQR)	18 (2–76)
90-d mRS 0–2, n (%)	17 (32.7)

ASITN/SIR, American Society of Interventional and Therapeutic Neuroradiology/Society of Interventional Radiology, ASPECTS, Alberta Stroke Program Early CT Score, DBP, diastolic blood pressure, HI, hemorrhagic infarction, ICA, internal carotid artery, IQR, interquartile range, IV, intravenous, MCA, middle cerebral artery, mRS, modified Rankin Scale, mTICI, modified Thrombolysis in Cerebral Infarction, NeuroICU, neurological intensive care units, NIHSS, National Institute of Health Stroke Scale, PH, parenchymal hemorrhage, SBP, systolic blood pressure, SD, standard deviation, SICH, symptomatic intracranial hemorrhage, TIA, transient ischemic attack, tPA, tissue plasminogen activator

**Fig. 3:**
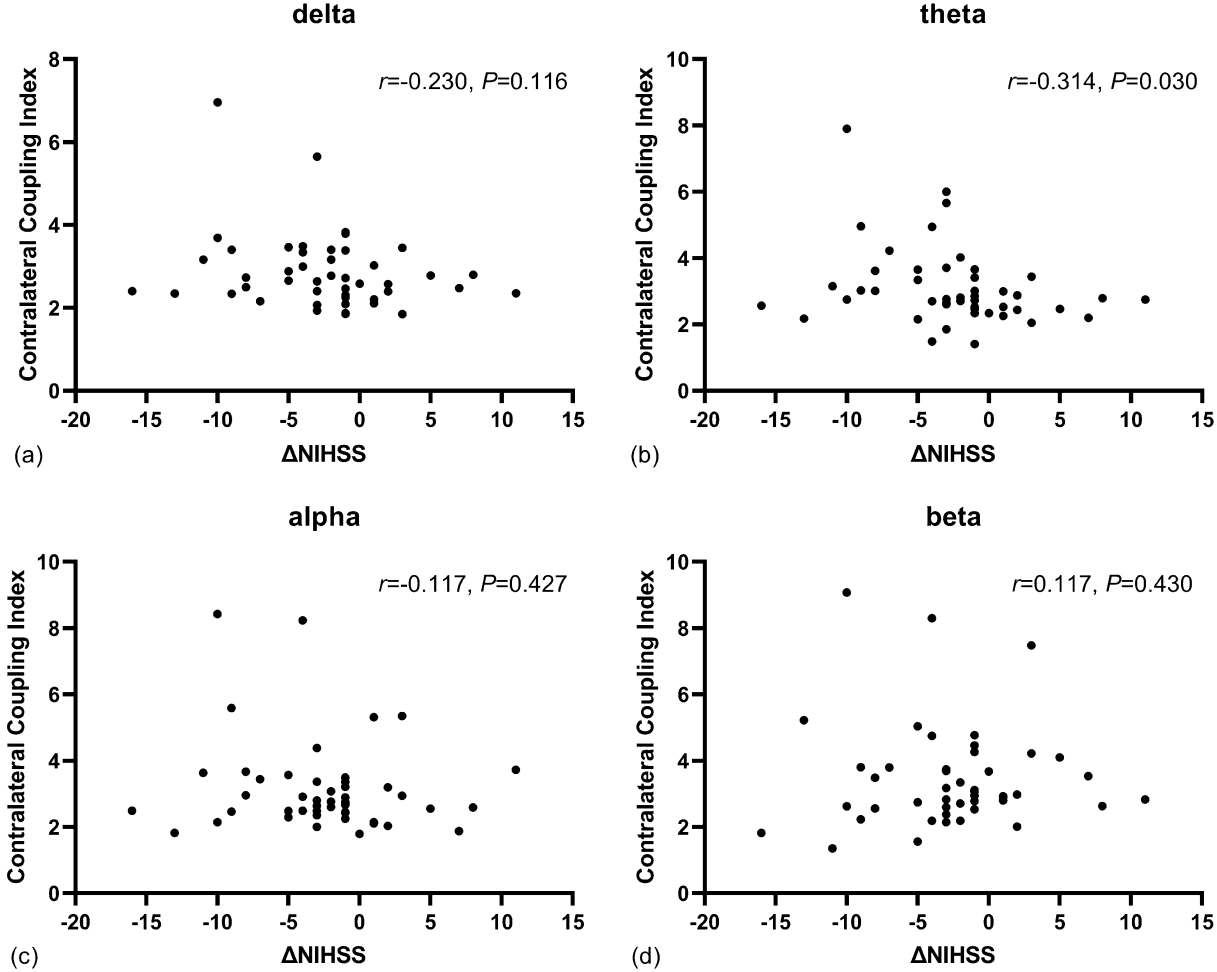
The association between ΔNIHSS and contralateral coupling indices across EEG frequency bands. Only the contralateral coupling in theta band was correlated with neurologic improvement at 7 days. EEG, electroencephalography, NIHSS, National Institute of Health Stroke Scale

**Fig. 4:**
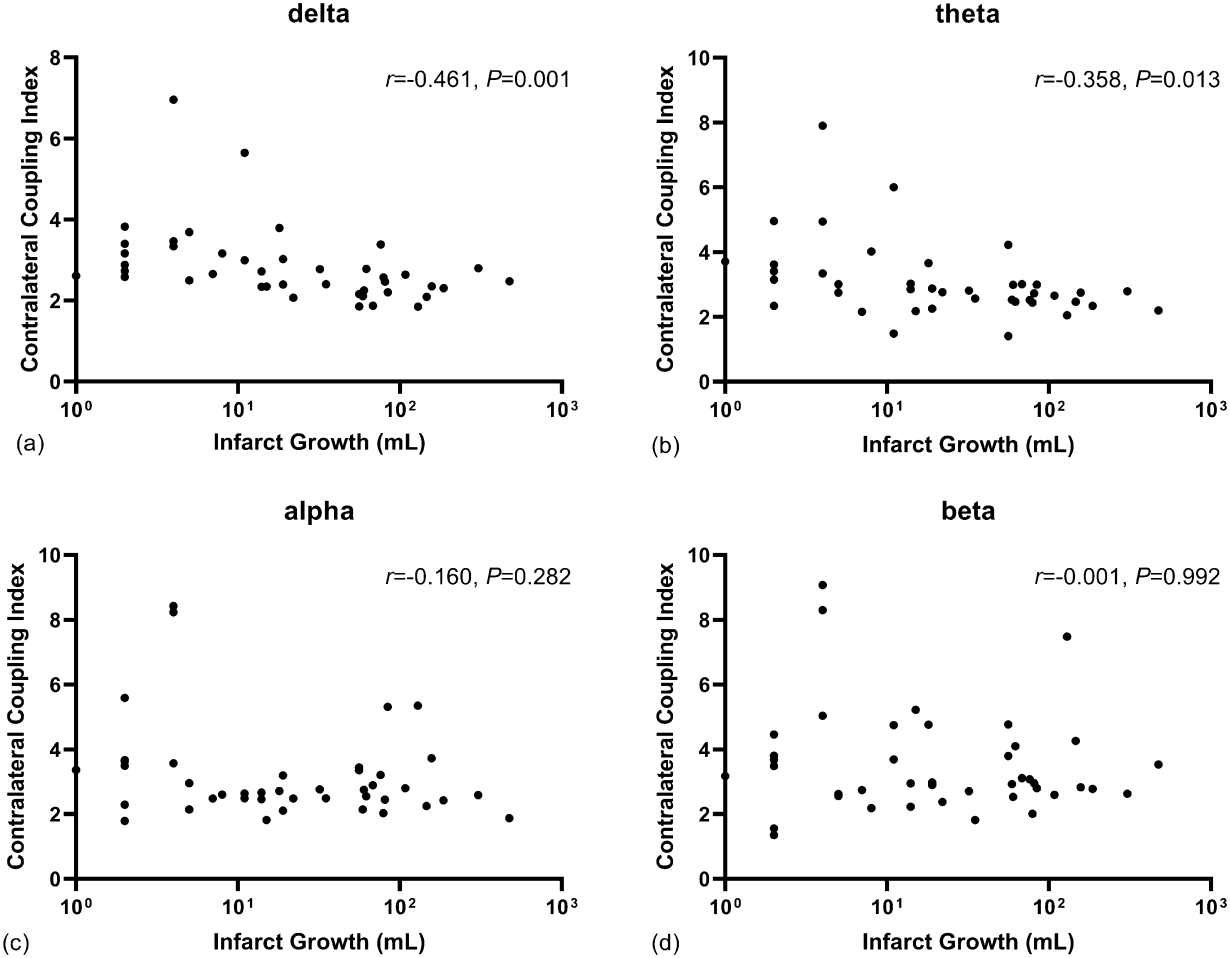
The association between infarct volume progress and contralateral coupling indices across EEG frequency bands. Both the contralateral coupling in delta and theta band was correlated with infarct progress at 7 days. Of note, five study participants with no infarct growth could not possibly present in the figures. EEG, electroencephalography

The median of ΔNIHSS was − 3 (IQR − 5 to − 1). ΔNIHSS was associated with contralateral coupling in theta bands (*P* = 0.030), whereas no significant correlation was found in delta, alpha, and beta bands (all *P* > 0.05) (Fig. 3). The median volume of infarct growth at 7 days was 18 (IQR 2–76) mL. The infarct growth was associated with contralateral coupling in delta (*P* = 0.001) and theta bands (*P* = 0.013). No significant association was noticed in alpha and beta bands (all *P* > 0.05) (Fig. 4).

A total of 32.7% (17/52) of patients had favorable outcomes at 90 days. Compared with the study participants with poor outcomes, those who achieved favorable outcomes at 90 days had a significantly higher contralateral coupling index in delta and theta bands, and a higher ADR on the stroke side (3.16 [2.40–3.47] versus 2.57 [2.21–2.80], *P* = 0.017; 3.34 [2.70–4.02] versus 2.73 [2.44–3.00], *P* = 0.044; 1.69 [1.00–3.72] versus 0.94 [0.46–1.46], *P* = 0.032, respectively) (Table 2). After adjusting for age, preprocedural NIHSS scores, ASPECTS, mTICI scores, and stroke volume at 24 h after EVT, stronger contralateral coupling in both delta and theta bands were independent predictive factors for favorable outcomes (adjusted OR for delta band 7.53 [95% CI 1.13–50.30] *P* = 0.037, adjusted OR for theta band 6.36 [95% CI 1.09–37.01] *P* = 0.039) (Table 3).

**Table 2:** Comparison of neurovascular coupling indices, ADR, and Mx between functional outcomes at 90 days (median [IQR])

Variables		Favorable outcome (*n* = 17)	Poor outcome (*n* = 31)^a^	Total (*n* = 48)^a^	*P* value
Contralateral coupling	Delta	3.16 (2.40–3.47)	2.57 (2.21–2.80)	2.63 (2.34–3.25)	0.017*
	Theta	3.34 (2.70–4.02)	2.73 (2.44–3.00)	2.77 (2.46–3.43)	0.044*
	Alpha	2.94 (2.48–3.66)	2.66 (2.35–3.19)	2.73 (2.44–3.40)	0.158
	Beta	3.48 (2.22–4.46)	2.95 (2.63–3.69)	2.96 (2.61–3.95)	0.690
Ipsilateral coupling on stoke side	Delta	2.91 (2.16–3.98)	2.56 (2.20–2.83)	2.65 (2.17–3.31)	0.286
	Theta	2.90 (2.53–3.58)	2.80 (2.23–3.15)	2.86 (2.31–3.35)	0.215
	Alpha	2.91 (2.37–4.05)	2.71 (2.43–3.04)	2.77 (2.40–3.47)	0.643
	Beta	3.48 (2.44–5.01)	3.14 (2.45–3.96)	3.14 (2.45–3.96)	0.612
Ipsilateral coupling on healthy side	Delta	2.71 (2.26–3.05)	2.83 (2.44–3.07)	2.74 (2.42–3.06)	0.821
	Theta	2.48 (2.15–3.61)	2.89 (2.40–3.25)	2.89 (2.25–3.45)	0.923
	Alpha	3.13 (2.36–4.09)	2.70 (2.37–3.39)	2.81 (2.36–3.66)	0.258
	Beta	2.96 (2.34–4.36)	2.90 (2.37–3.65)	2.91 (2.36–3.73)	0.872
ADR	Stroke side	1.69 (1.00–3.72)	0.94 (0.46–1.46)	1.13 (0.64–2.04)	0.032*
	Healthy side	2.37 (1.93–4.35)	1.71 (1.02–2.60)	2.10 (1.04–3.46)	0.215
Mx	Stroke side	0.0412 (0.0140–0.0596)	0.0471 (0.0197–0.0874)	0.0443 (0.0171–0.0815)	0.568
	Healthy side	0.0278 (0.0137–0.0498)	0.0373 (0.0194–0.0675)	0.0360 (0.0144–0.0639)	0.497

^*^ *P* < 0.05. ADR, alpha-to-delta ratio, EEG, electroencephalography, IQR, interquartile range, Mx, mean velocity index

^a^Four study participants were excluded from the calculation due to poor signal quality

**Table 3:** Association of contralateral coupling indices across four EEG frequency bands with favorable outcome at 90 days

Contralateral coupling	OR	95% CI	*P* value	aOR	95% CI	*P* value
Delta	2.08	0.93–4.62	0.074	7.53	1.13–50.30	0.037*
Theta	2.19	1.03–4.67	0.042*	6.36	1.09–37.01	0.039*
Alpha	1.96	0.94–4.06	0.071	2.03	0.72–5.76	0.182
Beta	1.43	0.78–2.61	0.244	1.19	0.45–3.17	0.725

aOR: adjusted for age, preprocedural NIHSS, ASPECTS, mTICI score and stroke volume at 24 h. aOR, adjusted odds ratio, ASPECTS, Alberta Stroke Program Early CT Score, CI confidence interval, EEG, electroencephalography, mTICI, modified Thrombolysis in Cerebral Infarction

A total of 61.5% (32/52) patients had a complete aCoW. No difference in contralateral coupling across the four EEG bands was found between complete and incomplete aCoW subgroups (all *P* > 0.05) (Table 4). In the complete aCoW subgroup, we failed to observe a stronger contralateral coupling in patients with favorable outcomes at 90 days. In contrast, in the incomplete aCoW subgroup, a statistically significant difference in the contralateral coupling in the delta band was observed between patients with favorable outcomes and those with poor outcomes at 90 days (3.16 [2.66–3.45] versus 2.40 [2.09–2.72], *P* = 0.004) (Table 4).

**Table 4:** Comparison of contralateral coupling indices between aCoW subgroups (median [IQR])

Subgroups	Complete aCoW				Incomplete aCoW				*P*3 value
	Favorable (*n* = 10)	Poor (*n* = 20)^a^	Subtotal (*n* = 30)^a^	*P*1 value	Favorable (*n* = 7)	Poor (*n* = 11)^a^	Subtotal (*n* = 18)^a^	*P*2 value	
Delta	3.03 (2.35–3.49)	2.63 (2.30–3.00)	2.69 (2.35–3.39)	0.403	3.16 (2.66–3.45)	2.40 (2.09–2.72)	2.58 (2.31–3.16)	0.004*	0.431
Theta	3.18 (2.57–3.62)	2.74 (2.53–3.00)	2.75 (2.53–3.41)	0.356	3.44 (2.75–4.96)	2.85 (2.34–3.00)	2.87 (2.34–3.44)	0.066	0.983
Alpha	3.20 (2.46–3.66)	2.73 (2.48–3.14)	2.76 (2.48–3.36)	0.403	2.94 (2.48–4.38)	2.66 (2.25–3.19)	2.63 (2.25–3.63)	0.363	0.595
Beta	3.97 (2.22–5.22)	2.94 (2.61–3.74)	3.01 (2.59–4.46)	0.379	2.74 (2.18–3.80)	2.95 (2.78–3.67)	2.96 (2.62–3.74)	0.643	0.496

^*^ *P* < 0.05. The *P*1 and P2 values represent the significant level of the comparison between outcomes within the aCoW subgroups, respectively. The *P*3 value represents the significant level of the comparison between the aCoW subgroups

aCoW, anterior Circle of Willis, IQR, interquartile range

^a^Study participants were excluded in each subgroup from the PACFC calculation due to poor signal quality

## Discussion

In this study of 52 patients with LVO strokes undergoing EVT, we measured the PACFC between the stroke-side EEG and the contralesional CBFV in 48 patients. Stronger contralateral coupling correlated with better neurological recovery and less stroke progress at 7 days. For the entire cohort, higher contralateral NVC indices in delta and theta bands were associated with improved odds of favorable functional outcome at 90 days, after adjusting for key prognostic covariates in stroke.

The mechanisms underlying futile revascularization and the early prediction of this outcome following EVT are poorly understood. Preprocedural collateral flow, assessed through digital subtraction angiography, CT angiography, and magnetic resonance angiography [17], significantly influences EVT effectiveness [18–21]. However, salvaged penumbral tissue remains vulnerable to ischemia after EVT due to microvascular dysfunction. Indicators such as failure to achieve a 30% or greater reduction in perfusion-weighted imaging lesion volume [22] or a decrease of at least 10 mL, as well as low relative transit time heterogeneity in perfusion MRI [23], predict the no-reflow phenomenon. Although neuroimaging techniques cannot fully assess whether the metabolic needs of reperfused brain tissue are met by the blood flow, NVC may offer insight into this physiological process.

Previously, we proved it was EEG that drove changes in CBFV using Granger causality analysis [12]. Our nonlinear PACFC algorithm provides spatial resolution, finding the NVC ipsilateral to the stenotic MCA was impaired [11]. Our work suggested that NVC assessment offers a noninvasive approach to understanding cerebral hemodynamic compensation after stroke, potentially aiding in the prediction of neurological recovery and functional outcomes after EVT. By quantifying coupling between EEG and blood flow metrics, NVC provides insights that complement traditional imaging, especially in cases with incomplete collateral pathways. However, its clinical utility and generalizability require validation in larger, diverse populations and the integration of arterial pressure monitoring for a fuller representation of cerebral hemodynamic function.

In this study, we separately calculated the NVC across four EEG frequency bands, finding that contralateral NVC in the delta and/or theta bands were significantly correlated with outcomes, possibly due to the difference in the predominant rhythms between two hemispheres after stroke. Cerebral ischemia is known to increase slow activity and decrease fast activity of EEG [24], disrupting higher-frequency EEG-CBFV coupling regardless of EEG amplitude. Notably, in our approach, the NVC values reflect EEG’s phase-amplitude coupling with CBFV, not EEG amplitude. Therefore, this change likely stems from a disruption in the concentration of EEG amplitudes at specific phases of CBFV, leading to a decrease in NVC at these higher frequencies. Adjusting for 24-h infarct volume in the logistic regression ruled out stroke burden as a driver of contralateral NVC. Moreover, represented by Mx, the cerebral autoregulation between the two hemispheres were not different in this cohort, suggesting higher contralateral coupling indices were not caused by a better autoregulation on the contralesional side.

The mechanism of contralateral NVC may rely on leptomeningeal collateral pathway. As the most detectable route, aCoW connects the bilateral proximal arteries of the anterior circulation. Interestingly, we found that stronger contralateral coupling was associated with better outcomes in patients with incomplete, rather than complete, aCoW. This paradox can be explained by the presumption that in patients lacking primary collaterals, contralesional blood flow might compensate for the supply to the ischemic tissue through leptomeningeal and other anastomoses [25], making contralateral NVC more crucial for prognosis. Because the aCoW is incomplete in nearly one fifth population [16, 26], NVC assessment can be a beneficial complement to radiological evaluation. However, the small sample limits conclusions on whether the link between contralateral NVC and outcomes is primarily due to aCoW status, warranting larger studies to clarify this relationship.

We did not identify a statistically significant difference in ipsilateral NVC between the stroke-affected and contralesional hemispheres, which may be attributed to methodological constraints. Because NVC was assessed in a resting state, the capacity to modulate blood flow may not have been fully captured without task stimulation, particularly given the short sampling time. Additionally, blood flow and/or NVC in the contralesional hemisphere may also be compromised. Although study participants with bilateral infarcts were excluded, the absence of NVC measurements in healthy controls limits our ability to confirm normal contralesional NVC. These issues could be addressed by expanding the sample size, increasing the sampling duration, enrolling healthy volunteers, and incorporating appropriate task stimulation.

There were several limitations in our study. First, the “spot” assessment of continuously changing NVC may lead to inaccuracy in predicting outcomes. The use of TCD made this limitation inevitable and potentially selected for patients with good acoustic windows. Second, our methodology could only link neuronal electrical activity and blood flow but could not determine the effect on flow velocity. Third, collaterals from posterior circulation, which also play a key role in compensation, were not evaluated in this study. Finally, the sample size was modest, making it impossible to adjust for variables such as the time gap between monitoring and recanalization, and the extent of preexisting atrophy or white matter lesions. Future studies should employ continuous CBF measurement methods, such as near-infrared spectroscopy, integrate arterial pressure assessments for comprehensive cerebral hemodynamic analysis, and use algorithms such as Granger causality or transfer entropy to enhance the characterization of neurovascular coupling and refine clinically relevant descriptors.

## Conclusions

A better contralateral coupling between stroke-side EEG and contralesional-side CBFV after EVT is associated with favorable neurological outcomes, suggesting contralateral NVC analysis may be potentially helpful to the brain function assessment after successful recanalization. This finding requires replication with expanded cohort size, optimized monitoring methods, and a deeper understanding of underlying mechanisms before clinical translation.

## Supplementary Information

Below is the link to the electronic supplementary material.

Supplementary file1 (DOCX 222 KB)
