# Supplementary material for: Contralateral Neurovascular Coupling in Patients with Ischemic Stroke After Endovascular Thrombectomy
Source: Neurocrit Care. 2025 Jan 7;42(3):996–1006. doi: 10.1007/s12028-024-02178-w (PMC12137508; doi:10.1007/s12028-024-02178-w)
Supplement: Supplementary file 1 — Supplementary file1 (DOCX 222 KB) [file 12028_2024_2178_MOESM1_ESM.docx]

Supplementary Materials

**Description of PACFC calculation**

*Data preprocessing*

The data preprocessing was executed with meticulous attention to detail, ensuring the reliability and validity of the subsequent analyses. Initially, a clinical expert visually inspected the raw data, identifying and excluding severely noisy or unreliable data portions for further consideration. This step was crucial to ensure that the analyses were conducted on high-quality, reliable data, thereby enhancing the robustness and credibility of the findings. Following this initial quality assurance step, the EEG data were subjected to a series of preprocessing steps using the EEGlab toolbox. The data were filtered using a high pass filter at 60 Hz and a lowpass filter at 0.1 Hz, isolating the frequency bands of interest and minimizing the influence of extraneous noise and slow drifts. Subsequently, Artifact Subspace Reconstruction (ASR) was employed with a standard deviation parameter set to 20, aiming to attenuate artifact-related components while preserving genuine neural signals. The EEG data were then rereferenced to the common average to minimize reference-related biases and enhance spatial specificity. Concurrently, the cerebral blood flow velocity (CBFV) data were bandpass filtered between 0.05-0.15 Hz. This step was designed to isolate the hemodynamic signals pertinent to cerebral autoregulation and neurovascular coupling while attenuating the influence of respiratory-related fluctuations. To optimize computational efficiency and resource utilization, both the EEG and CBFV data were down sampled to 125 Hz following the initial preprocessing steps. This reduction in sampling rate was implemented to balance computational feasibility with data resolution, ensuring that the analyses remained both robust and computationally viable.

*Definition and Quantification of Phase-Amplitude Cross-Frequency Coupling (PACFC)*

Phase-amplitude cross-frequency coupling (PACFC) is a neurophysiological phenomenon in which the phase of oscillations at one frequency modulates the amplitude of oscillations at another, often higher, frequency. This phenomenon plays a crucial role in the study's exploration of neural communication and integration within various brain regions and networks. It facilitates coordinated neural activity and the transfer of information, contributing to our understanding of cerebral functionality.


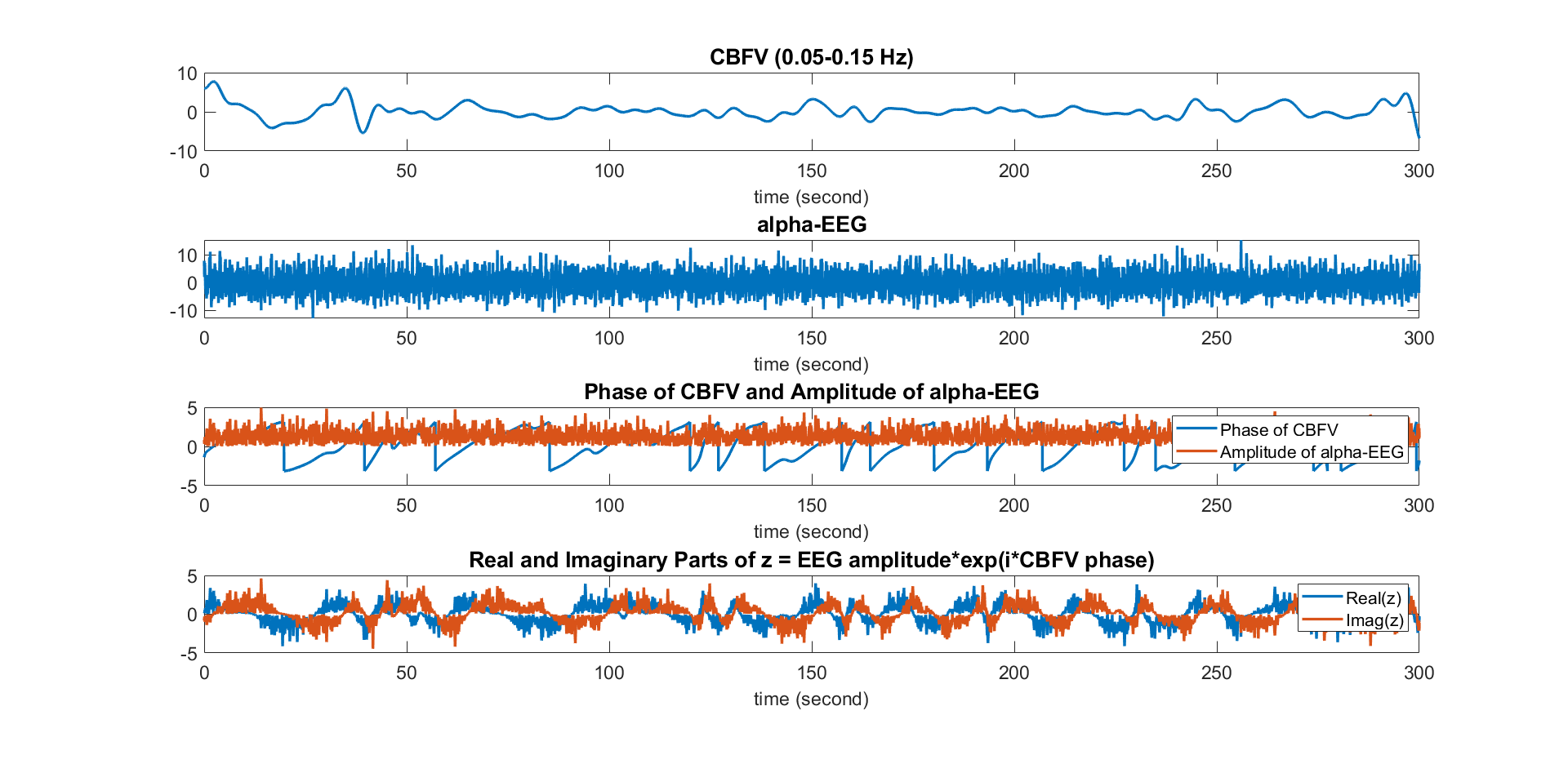


**Fig. S1** Steps involved in calculating PACFC between the phase of CBFV and the amplitude of alpha-EEG. The phase of the CBFV signal and the amplitude of the alpha-EEG signal were calculated using the Hilbert transform. Then, the complex-valued product of these two signals was obtained, and the PACFC was calculated using the magnitude of this product.

In the context of this study, two signals were analyzed: Signal $x(n)$, derived from electroencephalogram (EEG) recordings, is defined as$x(n)= A_{x}\left( n \right)e^{i\phi_{x}\left( n \right)}$, where $A_{x}\left( n \right)$ provides the amplitude in four distinct frequency bands: delta (0.5-4 Hz), theta (4-8 Hz), alpha (8-13 Hz), and beta (13-30 Hz), and $\phi_{x}\left( n \right)$ represents the phase. Whereas, signal $y(n)$ representing cerebral blood flow velocity (CBFV), is defined as $y(n)= A_{y}\left( n \right)e^{i\phi_{y}\left( n \right)}$, where $A_{y}\left( n \right)$ and $\phi_{y}\left( n \right)$ provide the amplitude and phase in the very low-frequency range (0.05-0.15 Hz), respectively.

The PACFC was quantified using the mean vector length method, computing the Modulation Index ($\rho_{MI}$) as follows:

$$\rho_{MI}=|\frac{1}{N}\sum_{n=1}^{N} A_{x}\left( n \right)e^{i\phi_{y}\left( n \right)}|$$

Where $\rho_{MI}$ is the modulation index, $N$is the number of data points, $A_{x}\left( n \right)$ is the amplitude of signal $x$(EEG) at time point $n$, $\phi_{y}\left( n \right)$is the phase of signal $y$ (CBFV) at time point $n$. **Fig. S1** visually presents the different steps of this method.


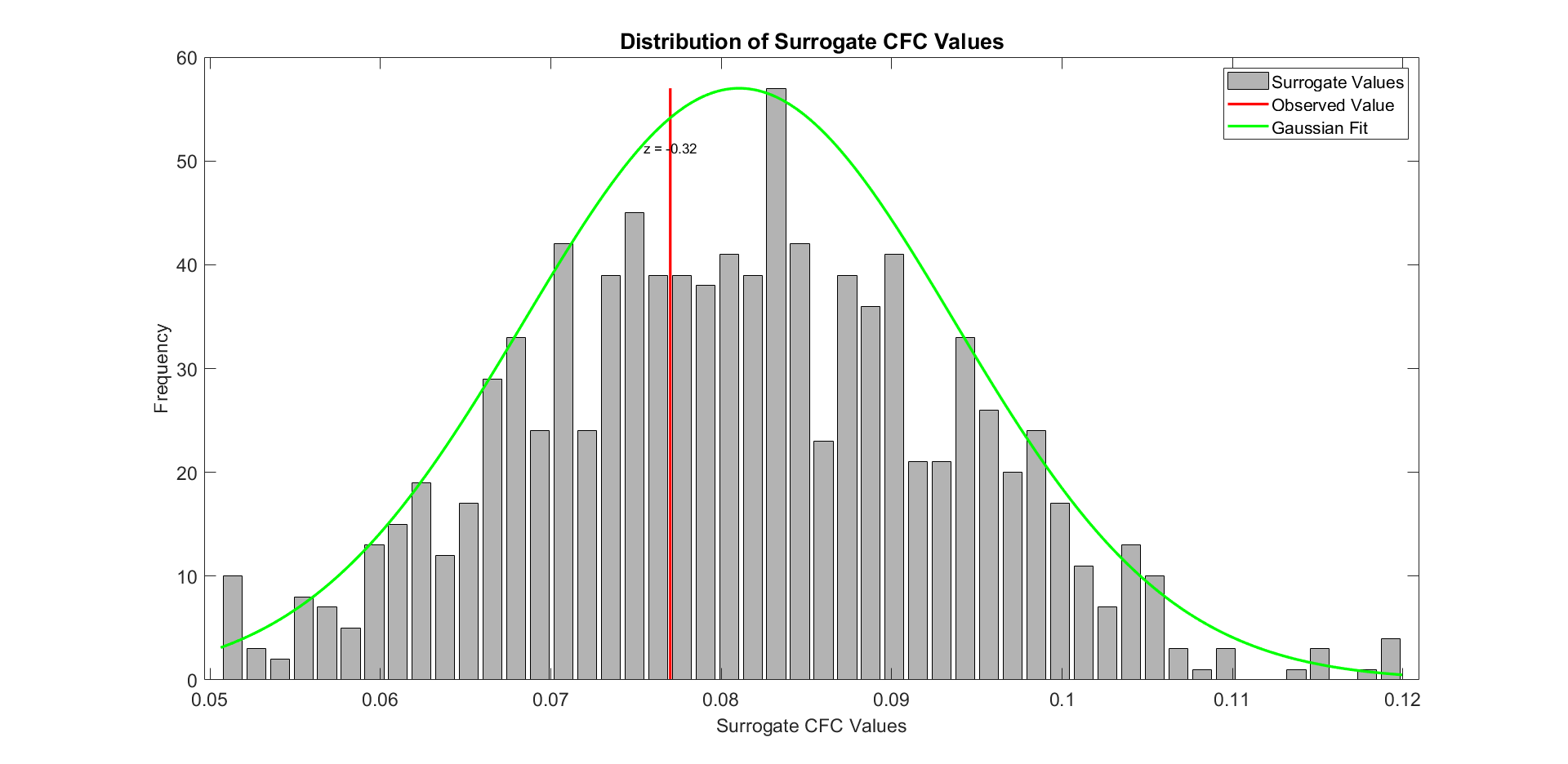


**Fig. S2** Example of surrogate normalization. The gray bars show the distribution of surrogate PACFC values, and the green curve is the fitted Gaussian distribution. The red line shows the originally calculated PACFC value (0.077), which was normalized to 0.32 (z-value) after surrogate normalization. The normalized PACFC value (z-value) was used for subsequent analysis.

To ensure the robustness and validity of the observed PACFC, a surrogate analysis method was utilized. This involved generating surrogate data to create a null distribution of $\rho_{MI}$values, followed by fitting a Gaussian distribution to the surrogate $\rho_{MI}$​ values to obtain the mean ($\mu$) and standard deviation ($\sigma$) of the surrogate distribution. The surrogate data were generated by cyclically shifting the amplitude of signal $x$ randomly, creating 200 surrogate datasets. The $\rho_{MI}$ for each surrogate dataset was computed using the aforementioned $\rho_{MI}$​ formula.

The Gaussian distribution fitting to the surrogate $\rho_{MI}$​ values is expressed as:

$$f\left( x | \mu,\sigma^{2} \right)=\frac{1}{\sigma\sqrt{2\pi}}e^{-\frac{1}{2}\left( \frac{x-\mu}{\sigma} \right)^{2}}$$

Where $f\left( x | \mu,\sigma^{2} \right)$ is the probability density function of the Gaussian distribution, $x$ is a variable, $\mu$ and $\sigma$ are the mean and standard deviation of the surrogate $\rho_{MI}$distribution, respectively.

Subsequently, the observed PACFC ($\rho_{MI}$​) was normalized using the mean and standard deviation derived from the surrogate distribution:

$$\rho_{NMI}=\frac{\rho_{MI}-\mu}{\sigma}$$

The process of surrogate normalization has been depicted in **Fig. S2**. This normalization not only ensures that the reported PACFC is statistically robust but also minimizes the effect of amplitude variations in the amplitude-providing signal $x$ (EEG). The amplitude of neural oscillations, particularly in EEG signals, can be influenced by numerous factors, including physiological conditions, cognitive states, and technical artifacts. Consequently, the PACFC, when calculated using methods like the mean vector length method, can be significantly impacted by these amplitude variations, potentially leading to spurious or biased results. This normalization, therefore, ensures that the normalized PACFC ($\rho_{NMI}$) is not only statistically validated against a null distribution but also adjusted for any systematic amplitude-related bias in the original signals.

It is worth highlighting a critical aspect of the data aggregation process in this study. Specifically, for each frequency band, the cross-frequency coupling (CFC) values from the three EEG channels on each side (stroke and healthy) were cumulatively combined before any statistical comparisons were conducted. This approach stemmed from the practical consideration that in real clinical settings, it is often challenging to pinpoint which specific EEG channel corresponds precisely to the location of a stroke or which channel might provide the most informative data regarding the affected area of the brain. Therefore, to account for this variability and uncertainty, CFC values from all available channels on the stroke and non-stroke sides were aggregated. Subsequently, the mean CFCs were calculated based on this aggregated data, comprising a total of 50 observations. The statistical comparisons were then performed on these mean CFC values. This methodological choice acknowledges the complexities of real-world clinical scenarios and ensures a more comprehensive and representative assessment of neural oscillatory activity in the context of stroke analysis.

In our analysis on NVC, we employed the mean vector length (MVL) method from Canolty *et al*. ^1^ for PACFC calculation. This method has its limitations such as sensitivity to the overall amplitude, impact from amplitude outliers, and the non-uniform distribution of phase angles. However, it's crucial to recognize that these limitations pertain primarily to raw PACFC values ^2^. To counter these issues and ensure more accurate PACFC assessments, we applied nonparametric permutation testing by means of surrogate normalization, a process that compares real PACFC values against those derived from surrogate data, thus nullifying the influence of amplitude and outliers. A similar approach was employed by Özkurt *et al.* ^3^ who used a statistically normalized modulation index after surrogate normalization. This additional step, as recommended by Özkurt *et al.* ^3^ and aligning with Cohen’s suggestions ^4^, adjusts for amplitude dependence in the MVL measure, thus providing a more robust and reliable analysis of the neurophysiological interactions in our study.

References

1. Canolty RT, Edwards E, Dalal SS, Soltani M, Nagarajan SS, Kirsch HE, et al. High gamma power is phase-locked to theta oscillations in human neocortex. *Science (New York, N.Y.)*. 2006;313:1626–1628.

2. Hülsemann MJ, Naumann E, Rasch B. Quantification of Phase-Amplitude Coupling in Neuronal Oscillations. Comparison of Phase-Locking Value, Mean Vector Length, Modulation Index, and Generalized-Linear-Modeling-Cross-Frequency-Coupling. *Frontiers in neuroscience*. 2019;13:573.

3. Özkurt TE, Schnitzler A. A critical note on the definition of phase-amplitude cross-frequency coupling. *Journal of neuroscience methods*. 2011;201:438–443.

4. Cohen MX. Analyzing Neural Time Series Data: Theory and Practice. Massachusetts, MA: The MIT Press.
